# A novel disorder reveals clathrin heavy chain-22 is essential for human pain and touch development

**DOI:** 10.1093/brain/awv149

**Published:** 2015-06-11

**Authors:** Michael S. Nahorski, Lihadh Al-Gazali, Jozef Hertecant, David J. Owen, Georg H. H. Borner, Ya-Chun Chen, Caroline L. Benn, Ofélia P. Carvalho, Samiha S. Shaikh, Anne Phelan, Margaret S. Robinson, Stephen J. Royle, C. Geoffrey Woods

**Affiliations:** 1 Cambridge Institute for Medical Research, University of Cambridge, Cambridge, CB2 0XY, UK; 2 Department of Paediatrics, College of Medicine and Health Sciences, United Arab Emirates University, Al-Ain, UAE; 3 Department of Paediatrics, Tawam Hospital, Al-Ain, UAE; 4 Max Planck Institute of Biochemistry, Department of Proteomics and Signal Transduction, Am Klopferspitz 18, 82152 Martinsried, Germany; 5 Neusentis, The Portway Building, Granta Park, Cambridge. CB21 6GS, UK; 6 Division of Biomedical Cell Biology, Warwick Medical School, Gibbet Hill Road, Coventry CV4 7AL, UK

**Keywords:** insensitivity to pain, clathrin, endosomal trafficking, neurogenesis

## Abstract

Congenital inability to feel pain is very rare but the identification of causative genes has yielded significant insights into pain pathways and also novel targets for pain treatment. We report a novel recessive disorder characterized by congenital insensitivity to pain, inability to feel touch, and cognitive delay. Affected individuals harboured a homozygous missense mutation in *CLTCL1* encoding the CHC22 clathrin heavy chain, p.E330K, which we demonstrate to have a functional effect on the protein. We found that CLTCL1 is significantly upregulated in the developing human brain, displaying an expression pattern suggestive of an early neurodevelopmental role. Guided by the disease phenotype, we investigated the role of CHC22 in two human neural crest differentiation systems; human induced pluripotent stem cell-derived nociceptors and TRKB-dependant SH-SY5Y cells. In both there was a significant downregulation of CHC22 upon the onset of neural differentiation. Furthermore, knockdown of CHC22 induced neurite outgrowth in neural precursor cells, which was rescued by stable overexpression of small interfering RNA-resistant CHC22, but not by mutant CHC22. Similarly, overexpression of wild-type, but not mutant, CHC22 blocked neurite outgrowth in cells treated with retinoic acid. These results reveal an essential and non-redundant role for CHC22 in neural crest development and in the genesis of pain and touch sensing neurons.

## Abstract (precis)

Congenital inability to feel pain is rare, but the identification of causative genes is translating into the development of novel analgesics. Nahorski *et al*. describe insensitivity to pain caused by mutations affecting the second clathrin heavy chain (CHC22), and reveal a role for CHC22 in pain and touch development.

## Graphical abstract

**Figure:**
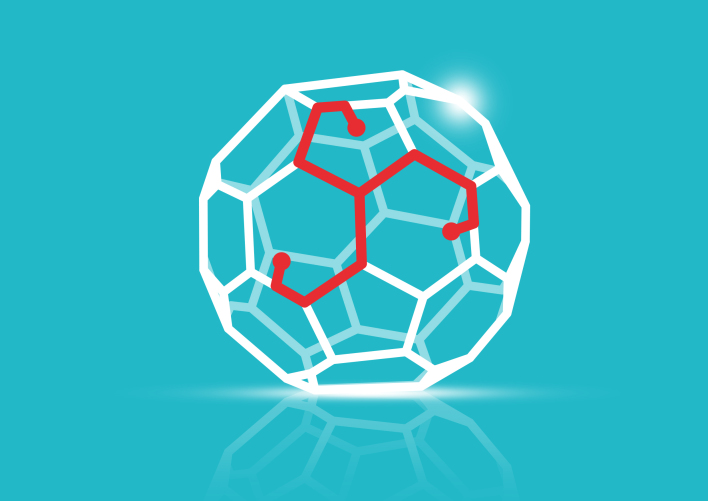
Congenital inability to feel pain is rare, but the identification of causative genes is translating into the development of novel analgesics. Nahorski *et al*. describe insensitivity to pain caused by mutations affecting the second clathrin heavy chain (CHC22), and reveal a role for CHC22 in pain and touch development.

## Introduction

Pain and touch are amongst the ubiquitous senses of complex multi-cellular organisms. For sight and hearing, mutations in <200 human genes are known to cause congenital blindness or deafness, respectively (Dror and Avraham, 2009; Daiger *et al.*, 2013). Study of these genes has substantially contributed to our understanding of development of these sensing systems. Far fewer genes have been discovered that cause a loss of pain sensing (Cox *et al.*, 2006; Rotthier *et al.*, 2012; Leipold *et al.*, 2013). However, the discovery of such genes is leading to treatments for individuals suffering from excess and unwanted pain—not a situation analogous to other senses (Holmes, 2012).

Pain is a complex sense, essential for detecting or warning of impending tissue damage and capable of detecting noxious stimuli of diverse origins and forms. Specialist nociceptors are of neuronal origin, and are the primary detectors of such insults. Mendelian disorders of painlessness, defined by the discovery of pathogenic mutations, can be clinically classified as developmental (a failure of nociceptor differentiation), dysfunctional (unresponsive nociceptors), and degenerative (where nociceptors prematurely die) (Cox *et al.*, 2006; Rotthier *et al.*, 2012; Leipold *et al.*, 2013). We have identified a novel Mendelian disorder characterized by a congenital insensitivity to pain, loss of touch sensation and severe cognitive delay, which clearly fits into the developmental category and hence defines a hitherto unrecognized human gene essential for pain perception and CNS development.

## Materials and methods

### Molecular genetics

#### Autozygosity mapping

Autozygosity mapping was undertaken using samples from the affected children as previously reported (Woods *et al.*, 2006*b*). In brief, genomic DNA from each affected individual was hybridized to the Affymetrix GeneChip^®^ Human Mapping 250 K Nsp Array. Results of the alleles present at each SNP for each individual were generated using Affymetrix software. Regions of shared allele-concordant homozygosity were sought using ExcludeAR (Woods *et al.*, 2004). We found five statistically significant shared allele-concordant homozygosity regions; chromosome 5:157–161 cM, chromosome 9:121–133 cM, chromosome 16:72–76 cM, chromosome 20:31–50 cM, and chromosome 22:17–26 cM. The finding of five shared homozygous regions encompassing 1.5% of the genome was within the expected result range given the consanguineous family structure (Woods *et al.*, 2006*a*). Analysis of the exome sequencing results detailed in the next section confirmed all of the homozygous regions identified, as the majority of contained DNA changes (>90% averaged over the five regions) were homozygous.

#### Exome sequencing

Exome sequencing performed on one affected child was undertaken using the SureSelect Human All Exon 50 Mb Kit (Agilent Technologies UK, Cat. No G3370A), enabling targeted capture of 50 Mb sequence of exonic regions and non-coding RNAs in the human genome. Sequencing was performed with the SOLiD^™^4 System (Applied Biosystems) with 50 bp fragment reads, to generate 4.3 Gb of sequence achieving 83% of bases coverage by >10 reads (sufficient for the detection of homozygous mutations) of the mappable targeted 50 Mb exome. Initially, the raw sequencing reads were mapped to the GRCh37 reference human genome and changes compared to this reference sequence identified (Bochukova *et al.*, 2012). We focused our analyses on non-synonymous coding, nonsense, splice site variants, and indels (insertions–deletions) involving exons. We filtered against: (i) known variations where the rare allele frequency was >1% (derived from dbSNP and 1000 Genomes); (ii) evolutionary conservation of the encoded amino acid, by use of the Human Genome Browser; and (iii) examination of the sequence reads containing potential mutations using the Integrated Genome Viewer (which shows misaligned, poorly sequenced and recurrent artefact changes). We did not filter on either the known function of recessive mutations in a variant-containing gene or on expression pattern. These filtering steps resulted in the reduction of 19 to only one potentially pathogenic variant in the concordant homozygous regions found in all affected children, c.988G>A in *CLTCL1* (p.E330K in CHC22). No further mutations in *CLTCL1* were found in a cohort of 39 painless patients of unknown genetic cause

#### Segregation analysis and control subject analysis

PCRs were designed to cover the region of *CLTCL1* containing the c.988G>A mutation, which was within exon 7. For this we used the Human Genome Browser and Primer3Web, and optimized the reaction in control human genomic DNA. Using genomic DNA samples from family members we found that the mutation was homozygous in all affected children, heterozygous in both parents, and heterozygous in the one unaffected sibling we could test. Using the same primers we analysed genomic DNA from a personally curated resource of 50 Arab and 130 Pakistani healthy adults. All were homozygous for the wild-type G allele of the mutation c.988G>A. The mutation was not present in the 1000 Genomes project. Details of websites and software used can be found in Appendix 1.

### Cell lines and culture conditions

SH-SY5Y cells were purchased from Sigma Aldrich, and were cultured in 100% Dulbecco’s modified Eagle medium (DMEM) supplemented with 10% foetal bovine serum, 100 U/ml penicillin-streptomycin (pen-strep) at 37°C and 5% CO_2_. HEK293 cells were maintained in 100% DMEM similarly supplemented. To achieve transient transfection of HEK293 cells, cells were plated into six-well plates at 2 × 10^5^ cells/well 24 h before transfection. Transfection was achieved using the *Trans*IT^®^-LT1 transfection reagent (Mirusbio) according to the manufacturer’s protocol. After 72 h the cells were harvested or fixed for western blot analysis or immunofluorescence analysis, respectively (see below).

To create stably expressing clones, cells were transfected as described for transient transfection. After 48 h, the cells were trypsinized and transferred to a 100 mm dish. After a further 24 h the media was changed for one containing 600 µg/ml G418 sulphate solution (Calbiochem). Once stably expressing colonies had formed, these were picked using cloning cylinders and successfully transfected clones determined by immunofluorescence and western blotting. Two successfully transfected CHC22.E330K clones were created, with all cells exhibiting a GFP signal by immunofluorescence and of the correct size determined by western blotting. However, wild-type clones proved harder to produce, with the best expressing clone (out of a further 20) being used for further experiments. This clone expressed comparable amounts of GFP per cell compared with the p.E330K expressing cells, but was not a pure population with a varying proportion of cells not expressing GFP (depending on passage number). These cells were routinely sorted for GFP-expressing cells before experimentation, but a totally pure population was not achieved. When these cells were subsequently analysed, only those cells expressing GFP were included in the analysis.

Transfection of siRNA was achieved using INTERFERin^®^ *in vitro* small interfering RNA (siRNA) transfection reagent (Polyplus) using a reverse transfection according to the manufacturer’s protocol. Briefly, cells were plated on Day 0 at 1.5 × 10^4^ cells/ml onto a mixture of 15 nM siRNA diluted in 200 µl Opti-MEM^®^ media and 12 µl INTERFERin^®^ reagent. Media was replaced on Day 3 with more of the same siRNA mixture added to the cells as on Day 0. Cells were imaged and assayed on Day 5. The methods for differentiating pain neurons from human pluripotent stem cells have been described previously (Chambers *et al.*, 2012).

Differentiation of SH-SY5Y cells has been described previously (Encinas *et al.*, 2000). Briefly, cells were plated at 1.5 × 10^4^ cells/ml. The following day media was changed for that containing 10 µM retinoic acid (or equal volume DMSO as control) and left for 5 days. Media was changed to fresh media containing retinoic acid on the third day. Cells were assayed at Day 5 after significant neurite outgrowth had occurred, but cells remained mitotic.

### DNA constructs, antibodies and RNA interference

Small interfering RNAs were purchased from Life Technologies or Qiagen. The siRNAs against CHC22 were GGCYCAAUCGUGAACUUCAtt (siCHC22.1) and GAAGAUGUUUGAUGACAUtt (siCHC22.2), CHC17 were CGUUGCUCUUGUUACGGAUtt (siCHC17.1) and GGGAAUUCUUCGUACUCCAtt (siCHC17.2) and scrambled was UUCUCCGAACGUGUCACGUtt.

Primary antibodies used include Human Clathrin Heavy Chain 2/CHC22 Polyclonal Sheep IgG Antibody (R&D Systems), Human Clathrin Heavy Chain 1/CHC17 Affinity Purified PAb (R&D Systems) (antibodies were proven not to cross-react with either endogenous or over-expressed protein; see Supplementary Fig. 2), Trk (pan) C17F1 Rabbit mAb 4609 (Cell Signalling Technology), anti-TrkB Mouse mAb 610 101 (BD Transduction Laboratories), GFP (D5.1) XP Rabbit mAb #2956 (Cell Signalling Technology), anti-beta Actin (AC-15) antibody (ab6276) (Abcam).

DNA constructs used are detailed in Fig. 2. DNA plasmids used to simultaneously knockdown endogenous CHC17 by RNA interference and to express GFP-tagged CHC22.WT or CHC22E330K under a CMV promoter, called ‘pBrain constructs’ have been described previously (constructs iii, iv, v), as have plasmids to expressed GFP-tagged CHC22 with no knockdown (construct i) (Royle *et al.*, 2005). The p.E330K mutation was introduced into plasmids i and v using the QuikChange^®^ II Site-Directed Mutagenesis Kit (Agilent Technologies) according to the manufacturer’s protocol, and successful plasmids confirmed by DNA sequencing (constructs ii and vi). Human CHC17 was knocked down using construct iv, which was otherwise the same as constructs v and vi, but did not express a protein fused to its GFP. Construct iii contained short hairpin (sh)RNA, was targeted against rat clathrin heavy chain and was unable to knockdown expression of human CHC17.

For making stably overexpressing SH-SY5Y cells, the GFP-tagged constructs i and ii described above were mutated to make them resistant to siCHC22.2 using the Site-Directed Mutagenesis Kit (Agilent Technologies). To achieve this, two site-directed mutagenesis reactions were set up to induce 4-bp changes that had no impact on the amino acids translated. The final sequence of the siRNA target region was as follows CTCCCAGCCCATGAA**A**ATGTT**C**GA**CC**GACATACCAGTCTG (bold and underlined represent mutated bases).

### Transferrin internalization assay

HEK293 cells plated onto poly-L-lysine-coated glass coverslips, were transfected as above with constructs which knock down endogenous CHC17 and re-express CHC22.WT or CHC22.E330K simultaneously (constructs v and vi). As a control for effective shRNA against CHC17 blocking transferrin internalization, constructs iii and iv were also transfected. On the third day after transfection, the cells were starved of serum for 35 min to deplete the cells of transferrin. The cells were then incubated with DMEM containing 100 µg/ml of Alexa Fluor^®^ 568-conjugated transferrin (Life Technologies) at 37°C for 10 min. Cells were then washed, fixed in 4% paraformaldehyde and mounted onto ProLong^®^ Gold antifade reagent with DAPI (Life Technologies). Cells were visualized with either a LSM510 or a LSM710 confocal microscope. For quantitative experiments, identical laser power and acquisition settings were used. For analysis of transferrin uptake, images were thresholded using ImageJ (NIH) software, the outline of each cell (using the green signal traced) around and the integrated density of the red signal (transferrin) calculated. Experiments testing the rescue of CHC17 depletion by either CHC22.WT or CHC22.E330K were carried out four independent times, with at least 136 and 153 cells counted for each condition, respectively. Experiments to confirm knockdown of CHC17 (using comparison of constructs iii and iv) caused significant reduction in transferrin uptake (previously confirmed by Hood and Royle, 2009; Willox and Royle, 2012) were carried out twice, with at least 58 cells counted for each condition.

Co-localization was measured by Pearson Co-localization Coefficient using Volocity Image analysis software.

### Producing heat maps from developmental transcriptome

The raw log2 transformed expression values (reads per kilobase of exon model per million mapped reads) of *CLTCL1* and *CLTC* extracted from various brain regions across development from embryo to mature adult brain, were downloaded from the ‘Brainspan Atlas of the Developing Human Brain’ (Website: ©2014 Allen Institute for Brain Science. BrainSpan Atlas of the Developing Human Brain [Internet]. Available from: http://brainspan.org/) (Miller *et al.*, 2014). These values were imported into Microsoft Excel and ordered according to developmental stage (time points with insufficient data were excluded from analysis at this stage). Where there were multiple samples for each developmental stage, an average expression value was calculated. Then, for each respective brain region, the expression values were calculated as a proportion of the largest expression value for that region (largest value 1) and a heat map produced with darker red colour assigned to values closest to 1 and dark blue representing the lowest expression value for that brain region. Hence, the heat map represents a spread of the highest to lowest expression of each given gene across development for each brain region, but the different regions themselves are not directly comparable with each other, nor can the colours for *CLTCL1* be compared directly to *CLTC*. The donor H376.VIII.53 for the 2-year time point was excluded from the analysis because for both *CLTCL1* and *CLTC* it represented a significantly outlying result, likely to represent an unknown issue with the donor or sample preparation and not to represent a genuine transcriptional level of either gene at this time point.

Previous studies have shown that *CLTC* is expressed at greatly increased amounts compared to *CLTCL1*, and so we sought to analyse the comparative amounts of the two genes throughout CNS development. The analysis described above allowed us to demonstrate the relative changes in the expression levels of the two genes over development, but not directly compare the two clathrins. To compare the levels of *CLTCL1* to *CLTC* directly we calculated the percentage of overall clathrin heavy chain expression represented by *CLTCL1* and *CLTC*, respectively. To achieve this, the log2 transformed expression values originally downloaded were reverse log transformed to comparative transcription values. The expression of *CLTCL1* expression as a percentage of total clathrin levels was calculated for each time point (percentage of *CLTCL1* expression compared to *CLTCL1* + *CLTC* expression), and the data split into either pre- or postnatal. The pre- and postnatal groups were then compared using Students *t*-test.

### Neurite outgrowth assays

SH-SY5Y cells were transfected with siRNA, as described above, and left for 5 days (change of media and second hit of siRNA added on Day 3). This time point was selected because a clear distinction in cell phenotype was apparent (Days 4 and 6 were also tested with comparable results), and knockdown was optimal. For each experiment, at least three fields of view were imaged for phase contrast images using an EVOS FLCell Imaging System and for phase contrast plus green signal for stably expressing cells. The images were blinded and neurite outgrowth measured using ImageJ software. For ‘neurite length per cell’ measurements, all neurites, however small, were measured and the total neurite length of all cells combined, divided by the number of cells in that field of view. For ‘percentage of cells bearing neurites’, the same data were analysed, but the number of ‘neurites’ taken as only neurites longer than twice the average size of a SH-SY5Y cell body at that time point.

### Glutathione S-transferase purification

Bovine clathrin heavy chain terminal domain plasmid (residues 1–363) has been described previously (Miele *et al.*, 2004). The p.E330K mutation was introduced into the sequence using QuikChange^®^ II Site-Directed Mutagenesis Kit (Agilent Technologies). Both wild-type and mutant plasmids were prepared as previously described to produce protein with an N-terminal GST (glutathione S-transferase) tag (Drake and Traub, 2001). Briefly, the GST fusion proteins were produced in *Escherichia coli* BL21 DE3 plysS cells. Log-phase cultures (A600 ∼0.8) growing at 37°C were placed at 22°C in a shaking incubator and induced to produce protein by addition of isopropyl-1-thio-β-D-galactopyranoside (IPTG) to a final concentration of 100 µM, and left overnight. The bacteria were then recovered by centrifugation at 4500 rpm at 4°C for 15 min and the cell pellets frozen at −80°C. GST fusions were collected on glutathione Sepharose 4B after lysis of the bacteria, and cleaved from the GST with thrombin. After extensive washing, lysates were eluted from the beads and the thrombin inactivated with ABESF [4-(2-aminoethyl) benzenesulphonyl fluoride hydrochloride]. Proteins were concentrated to ∼10 mg/ml and purified by gel filtration on an analytical S200 column (GE healthcare) in 200 mM NaCl, 10 mM HEPES pH 7.5, 1 mM dithiothreitol. Samples for circular dichroism at 0.5 mg/ml were dialysed into 50 mM Na phosphate pH 7.5.

### Protein extraction

Cells were grown in six-well plates, washed with phosphate-buffered saline (PBS) and scraped in RIPA buffer (Tris, pH 7.4, NaCl 150 mM, EDTA, 0.5 mM, 1% Triton), containing protease inhibitors (Roche Applied Sciences). Lysates were cleared by centrifugation at (12 000 rpm at 4°C for 20 min) and levels of total cellular protein tested using the DC^™^ protein assay kit according to manufacturer’s instructions (Bio-Rad).

### Protein blotting

Fifteen to twenty-five micrograms of extracted total cellular protein were separated using either 8% SDS-page gels or NuPAGE^®^ Novex 3–8% Tris-Acetate Protein Gels. Primary antibodies are described above. Secondary antibodies were purchased from Dako and signal detected using the enhanced chemiluminescence (ECL, Amersham) western blot analysis system.

## Results

### Identification of a p.E330K mutation in *CLCTL1* in a consanguineous, painless family

**Figure 1:**
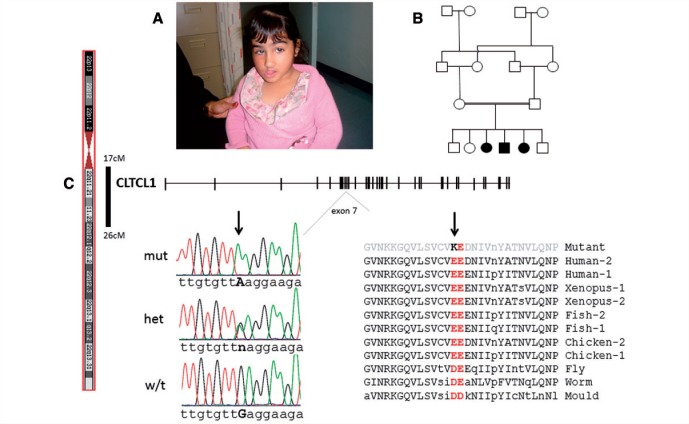
**Discovery of a *CLTCL1* mutation in individuals with lack of pain or touch sensing and severe intellectual disability.** (**A**) Image of an affected child aged 5 years, noting corneal opacity secondary to lack of a corneal reflex. (**B**) Family pedigree with parents being double first cousins and the filled in symbols indicating their affected children (two female and one male). (**C**) The affected individuals were concordant and homozygous for consecutive variants in five autosomal regions. The candidate mutation was localized to one of these regions on chromosome 22. This contained the gene *CLTCL1* for which a cartoon of the exon/intron structure is shown. In exon 7 we identified the c.988G>A mutation in *CLTCL1*, for which electrophoretograms of the Sanger sequencing results are shown for an affected child homozygous for the mutant A allele, an unaffected carrier parent heterozygous for the wild-type G and mutant A alleles, and a typical control wild-type G allele. The gene mutation resulted in the alteration of an invariant negatively charged glutamic acid 330 to a basic charged lysine in the encoded protein CHC22. The amino acid at position 330 in CHC22 is the first of an invariant pair of acidic amino acids present in all clathrin heavy chain proteins, which is evolutionarily conserved through vertebrates (with two clathrin heavy chains, CHC17 and CHC22, termed 1 and 2, respectively in this figure), insects and amoeba (with a single CHC17 like clathrin heavy chain). Protein sequence data are for human as a representative mammal (CHC22 is NP_009029.3; CHC17 is NP_004850.1), *Xenopus tropicalis* for amphibians (XP_002931934.2; NP_001011039.1), *Danio rerio* for fish (XP_005172491.1; XP_005157480.1), *Gallus gallus* for birds (Human Genome Browser, BLASTp and ACA13364.1; NP_001073586.1), *Drosophila melanogaster* for flies (NP_477042.1), *Caenorhabditis elegans* as a nematode (NP_499260.1), and *Dictyostelium discoideum*, a slime mould, as an amoeba (XP_642717.1). Upper case amino acid nomenclature is for invariance within a class, lowercase for amino acids that vary between species of the class.

We ascertained a consanguineous family of Balochi origin after the birth of their second child—subsequently a third affected child was born (Fig. 1A). All were unable to sense pain from birth and were unresponsive to soft touch. By 3 months it was apparent that they were developmentally delayed and in those >2 years it became clear that they had severe, non-progressive learning difficulties. Motor movements and strength were normal and hot and cold could be perceived. Thus the phenotype was novel and distinct from known causes of Mendelian congenital painlessness such as hereditary sensory and autonomic neuropathy type IV or V (Rotthier *et al.*, 2012). For further clinical details, see the online Supplementary material.

To find the causative gene mutation, autozygosity mapping was performed and the results analysed on the assumption of autosomal recessive inheritance. Using Affymetrix SNP chips we delineated five shared homozygous and concordant regions in the affected family members—comprising a total of 49 cM (1.5% of genome). Exome analysis was performed on one affected child and analysis targeted to the five regions identified. Only one candidate mutation was identified, c.988 G>A in *CLTCL1*, which was homozygous, previously unidentified, and potentially pathogenic (Fig. 1C and Supplementary material). The *CLTCL1* mutation segregated in the family as expected for an autosomal recessive, with all three affected individuals homozygous for the mutation, the parents both heterozygous and the one unaffected sibling available for analysis, also heterozygous. Furthermore, we did not find the mutation in 360 control chromosomes or in the 1000 Genomes project (2012). We found no other potentially pathogenic, concordant, homozygous or biallelic mutations in the affected children.

*CLTCL1* encodes the minor clathrin heavy chain (CHC22), a relatively understudied protein proposed to be involved in intracellular endosome trafficking (Vassilopoulos *et al.*, 2009). The common clathrin heavy chain (CHC17) is encoded by the gene *CLTC* on chromosome 17. CHC17 is expressed at a far higher level than CHC22 and is present ubiquitously in all cell types; however, the pattern of CHC22 expression differs between tissues and during development (Kedra *et al.*, 1996; Vassilopoulos *et al.*, 2009). The mutation identified in *CLTCL1* leads to the substitution of an invariant glutamic acid to a lysine, p.E330K, which is positioned at the C-terminus of the seven bladed WD repeat containing β-propeller that forms the adaptor binding domain of the clathrin heavy chains. Position 330–331 in the clathrin heavy chain is occupied by a pair of amino acids with negatively charged side chains (predominantly glutamic acid, less often aspartic acid). This pair of amino acids is invariant in both clathrin heavy chain proteins in all mammals (with the exception of rodents that have no *CLTCL1* gene), fish, amphibians, birds, and the phylogenies with a single *CLTC* gene, e.g. insects, worm and slime mould (Fig. 1C).

### p.E330K renders CHC22 non-functional in rescuing endocytosis at the cell membrane

**Figure 2:**
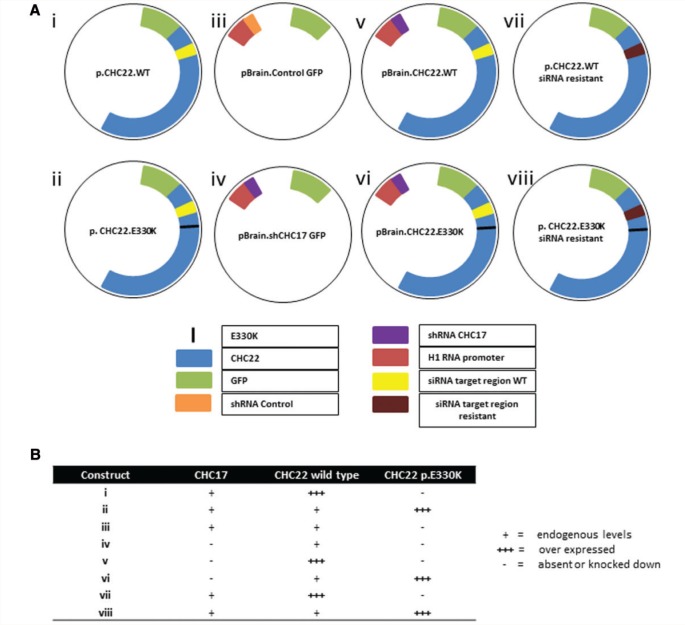
**DNA plasmid constructs used in this study.** (**A**) The eight constructs used in this study, colour coded for each feature. (**B**) Summary of the effects of transfection of each construct (i–viii) on CHC17 and CHC22 level.

**Figure 3:**
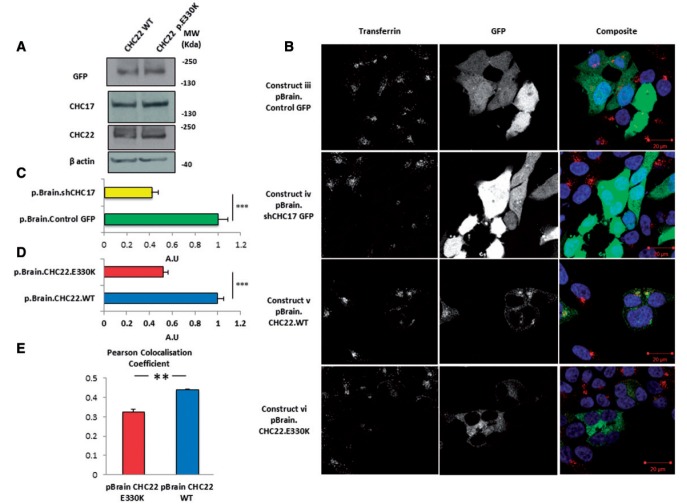
**p.E330K in *CLTCL1* is a pathogenic mutation**. (**A**) The p.E330K mutation has no effect on the steady state levels of CHC22 or CHC17 in HEK293 cells determined by western blotting. (**B**) Transferrin internalization assay was used to determine the functionality of the p.E330K mutant in HEK293 cells. Red signal represents transferrin-Alexa Fluor^®^ 546, green indicates GFP or CHC22.GFP, and blue DAPI. (**C**) Cells transfected with control shRNA and tagged with GFP (construct iii) display normal transferrin internalization, whereas transfection with shRNA against CHC17 (construct iv) significantly inhibited transferrin internalization (*P* < 0.001). (**D**) Cells transfected with the same shRNA construct against CHC17 also expressing wild-type CHC22 (construct v) displayed functional transferrin internalization, whereas cells transfected with the same construct except carrying the p.E330K mutation (construct vi) demonstrated no rescue of transferrin internalization (*P* < 0.001 compared with pBrain.CHC22.WT). (**E**) There was also a reduced co-localization of CHC22.E330K (construct vi) with fluorescent transferrin compared with CHC22.WT (construct v) tested by Pearson Co-localization Coefficient (*P* < 0.005). (**C**) Represents the pooled data from two independent repeats confirming the previously demonstrated effect of CHC17 knock down on transferrin internalization (Esk *et al.*, 2010; Willox and Royle, 2012). At least 58 cells counted for each transfection condition. (**D**) Represents the pooled data from four independent experiments normalized to pBrain.CHC22.WT. At least 136 cells counted for each transfection. (**E**) Represents pooled data from three independent experiments. Statistical tests were two-tailed Student’s *t*-tests. Error bars represent standard error. For explanation of the constructs used in greater detail, see Fig. 2.

To assess the functional significance of the p.E330K mutation, it was introduced into a previously reported, functional N-terminal GFP-tagged CHC22 construct (constructs i and ii) (Hood and Royle, 2009) (Fig. 2). There was no difference in the steady state levels of mutant CHC22 versus wild-type CHC22 when expressed in HEK293 cells, nor did this affect the stability of CHC17 (Fig. 3A and B). Further, when produced in *E. coli*, we found that the mutation did not affect folding of the clathrin N-terminal β-propeller domain (Supplementary Fig. 1).

We sought to investigate CHC22 endocytic function using the validated transferrin internalization assay. Transferrin receptors are internalized upon binding exogenous fluorescent tagged transferrin by CHC17-containing clathrin coated vesicles (CCVs). Knockdown of CHC17 blocks this process (Hood and Royle, 2009; Esk *et al.*, 2010). CHC22 has been shown to be able to substitute for CHC17 function and mediate CCV-dependent transferrin internalization, but only when expressed at high levels (comparable to wild-type CHC17 expression levels) and in the absence of endogenous CHC17 (Liu *et al.*, 2001; Towler *et al.*, 2004; Hood and Royle, 2009; Esk *et al.*, 2010; Brodsky, 2012). We therefore used this assay to test if this mutation affects CHC22 CCV function *in vivo* using constructs either expressing wild-type or p.E330K containing CHC22, while simultaneously knocking down endogenous CHC17 (constructs v and vi; Fig. 2) (Royle *et al.*, 2005; Hood and Royle, 2009). We verified our methodology by showing significant reduction in the uptake of fluorescent transferrin in cells treated with CHC17 shRNA compared to GFP control cells (transfection with constructs iii and iv; Fig. 3B and C). Constructs v and vi were transfected into HEK293 cells to produce cells lacking CHC17 and expressing GFP-tagged CHC22 (Fig. 3B). In these CHC17-deficient cells, over-expression of CHC22.WT was sufficient to induce significant transferrin endocytosis. However, the mutant CHC22.E330K was unable to rescue the deficiency of CHC17 (Fig. 3B and D). This was also reflected in reduced co-localization between mutant CHC22.E330K and transferrin compared with the wild-type construct (Fig. 3E). These data demonstrate that the p.E330K mutation disrupts the ability of CHC22 to rescue clathrin-mediated endocytosis, thus suggesting that the p.E330K is a pathogenic mutation.

### *CLTCL1* is highly expressed in the developing human brain and is downregulated in the postnatal cortex

**Figure 4:**
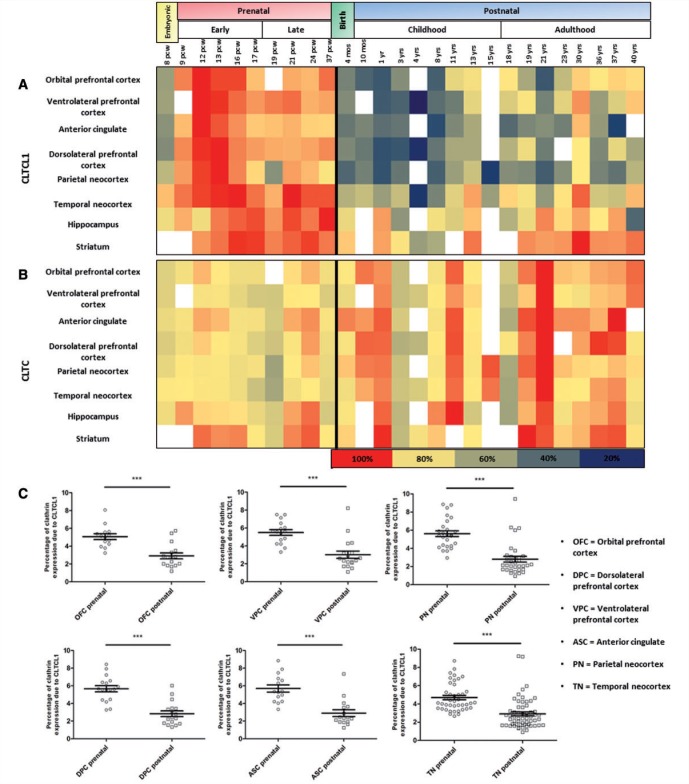
***CLTCL1* is highly expressed in the developing human cortex.** Heat maps demonstrating the expression profiles of *CLTCL1* (**A**) and *CLTC* (**B**), respectively, throughout development of various regions of the human brain. Note the increased expression of *CLTCL1* during early prenatal development and a drop after birth contrasting with the more linear expression profile for *CLTC*. The colours of the heat map are expression values, transformed to a log2 scale and calculated as a percentage of the highest expression time point for each brain region, respectively. The colour scale ranges from dark blue representing low expression through yellow to dark red representing high expression. The key below demonstrates the colours assigned to each percentage expression. Blank boxes represent where no data were available. (**B**) Graphs comparing the percentage of clathrin heavy chain expression (*CLTC* and *CLTCL1*) represented by *CLTCL1* in various regions of the cortex. All regions show a significant downregulation in the proportion of clathrin expression attributed to *CLTCL1* after birth. On average *CLTCL1* was between and 6% of total clathrin before birth and ∼3% after birth. Horizontal bars represent the mean and error bars, the standard error. Significance tests were Student’s *t*-test. Three stars represent *P* < 0.0001.

The expression of *CLTCL1* compared to that of *CLTC* is quite modest, with skeletal muscle displaying the highest expression of any tissue tested to date (Vassilopoulos *et al.*, 2009). We hypothesized that if *CLTCL1* were to play a neurodevelopmental role, its expression pattern might reveal an upregulation in the developing human brain. To address this question, we analysed transcriptional data previously published as part of the Developmental Transcriptome published by the Allen Institute (http://brainspan.org/). The gene expression data for both *CLTC* and *CLTCL1* were downloaded and analysed for the periods spanning from embryonic (9 weeks post-conception) until adult (40 years old). We identified that throughout all regions of the cortex analysed, *CLTCL1* displayed a peak of expression between 12 and 13 weeks post-conception, which then dropped between 4 - and 7-fold by early childhood (Fig. 4A). However, the expression of *CLTC* was consistent throughout prenatal brain development and at no developmental stage was up- or downregulated by >30% (likely indicating experimental variability, or variance between test subjects) (Fig. 4B). The hippocampus and striatum also displayed increased *CLTCL1* expression prenatally although peaking later in prenatal development (Weeks 17–21), also a less marked reduction of expression after birth (Fig. 4A). The thalamus and cerebellum did not show significantly increased *CLTCL1* expression prenatally (data not shown).

Next we transformed the relative expression data to determine the comparative expression levels of *CLTCL1* and *CLTC* during brain cortex development. The percentage of clathrin heavy chain represented by *CLTCL1* in the developing prenatal cortex was between 5 and 5.7% on average (Q1 4.3–4.6%, Q3 5.5–6.7%). After birth, this value significantly dropped to between 2.8 and 3.0% total clathrin heavy chain expression represented by *CLTCL1* (Q1 1.7–2.1%, Q3 3.1–3.6%) (Fig. 4B).

### CHC22 is downregulated in the progression from neural crest to sensory neuron-like in the development of a pain neuron

**Figure 5:**
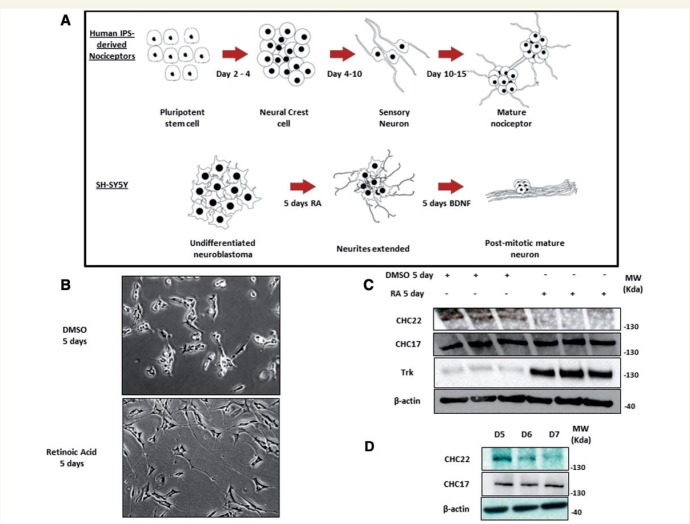
**CHC22 is downregulated upon differentiation from a neural crest type cell to a sensory neuron-like cell.** (**A**) Schematic representation of the differentiation of human induced pluripotent stem cells differentiated into pain neurons with small molecule inductors and NGFβ and the differentiation of SH-SY5Y neural crest derived neuroblastoma cells with all-trans retinoic acid (RA) and BDNF. (**B**) Representative phase contrast image of SH-SY5Y cells treated with retinoic acid or control DMSO (dimethyl sulphoxide) for 5 days; note the appearance of long neurites extending from the cell body in the retinoic acid treated cells. (**C**) CHC22 is significantly downregulated upon treatment with retinoic acid, whereas TRKB shows a significant upregulation under the same conditions. (**D**) CHC22 is downregulated across Days 5–7 of pain neuron differentiation from human pluripotent stem cells, occurring at the transition of neural crest to sensory neuron cells and induction of neurite outgrowth. For full time course, see Supplementary Fig. 3.

The phenotype of affected children harbouring the *CLTCL1* mutation indicates both a central and peripheral nervous system defect. Having found *CLTCL1* to be expressed at an increased level in the prenatal cortex and to show an expression profile suggestive of a neurodevelopmental role, we sought to define a possible role for CHC22 during neurogenesis. To achieve this we investigated the expression profile of CHC22 during peripheral nervous system development in culture. Both pain and touch sensing neurons originate from neural crest precursor cells (Liu and Ma, 2011). Pain sensing nociceptors predominantly express the transmembrane receptor TRKA (encoded by *NTRK1*), which is activated by the neurotrophin NGFβ (encoded by *NGF*) during development to induce differentiation to a post-mitotic nociceptor. Touch sensing neuron development is similarly dependent upon activation of transmembrane TRKB (encoded by *NTRK2*) by the neurotrophin BDNF (Chao, 2003; Marmigere and Ernfors, 2007). Therefore, we studied: human induced pluripotent stem cells differentiated into pain neurons with small molecule inductors and NGFβ (Chambers *et al.*, 2012); and human SH-SY5Y cells, which are neural crest derived and differentiate into neural cells following exposure to all-trans retinoic acid and BDNF (Kaplan *et al.*, 1993). The differentiation of these cells progresses from a stem-cell like morphology, to a mitotic cell exhibiting significant neurite outgrowth, and finally to a post-mitotic neuronal cell (Fig. 5A and B). In both systems induction of neuronal differentiation and neurite outgrowth was accompanied by a significant downregulation of CHC22 protein levels (Fig. 5C and D).

### CHC22 downregulation drives neurite outgrowth

**Figure 6:**
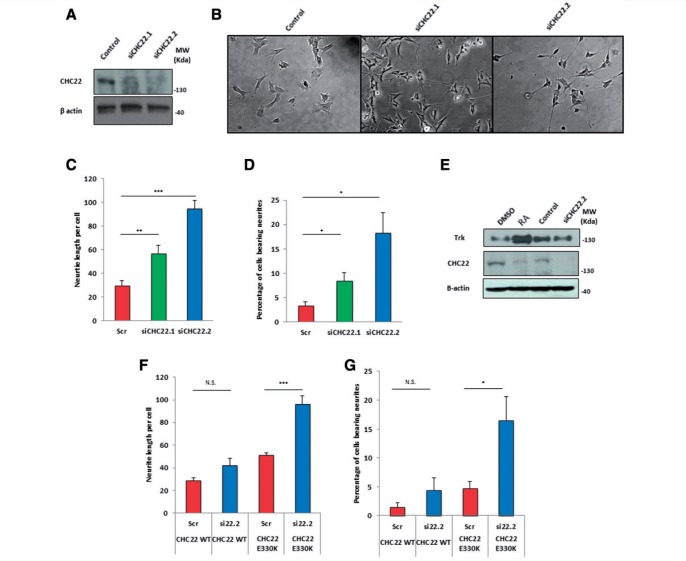
**Knockdown of *CLTCL1* causes induction of neurite outgrowth**. (**A**) Western blot demonstrating knockdown of CHC22 using two separate siRNAs. Images taken on Day 5, SH-SY5Y cells having been treated with 15 nM siRNA on Days 1 and 4. (**B**) Representative Day 5 images of cells treated with indicated siRNAs. Cells treated with siRNA against CHC22 show significant neurite outgrowth. (**C**) Quantification of average neurite length per cell and (**D**) percentage of cells bearing a neurite (at least twice the cell body length), pooled from six independent repeats of the experiment with at least 1500 cells counted for each condition. (**E**) Western blot of SH-SY5Y cells differentiated by 5-day retinoic acid treatment or transfection with CHC22 siRNA, both experiments lysed at Day 5. There is an upregulation of TRKB in the retinoic acid treated cells, which is not apparent in the siCHC22 treated cells. (**F**) No significant increase in neurite length per cell or (**G**) percentage of cells bearing neurites in SH-SY5Y cells overexpressing a siRNA resistant CHC22.WT GFP upon knockdown of endogenous CHC22. In p.E330K expressing cells there is a significant increase in both the neurite length and percentage of cells bearing a neurite in cells overexpressing CHC22.E330K, upon knockdown of endogenous CHC22. Statistical tests were two-tailed Student’s *t*-tests: *P < 0.05, **P < 0.01, ***P < 0.0001. Error bars represent standard error. N.S = not significant.

To test if the fall in CHC22 levels was involved in inducing neural crest cell differentiation or was a consequence of it, we designed two siRNAs against *CLTCL1*, both of which we confirmed to induce significant downregulation of CHC22 protein levels at Day 5 (Fig. 6A). SH-SY5Y cells treated with either siRNA against *CLTCL1* displayed a dramatic change in cellular morphology with a more neuronal phenotype characterized by significantly increased neurite length per cell and percentage of cells bearing a neurite (defined as greater than twice the cell body length) (Fig. 6B–D). We confirmed that the effects on neurite outgrowth were due to CHC22 knockdown and not off-target effects by rescuing these effects by over expression of CHC22 in SH-SY5Y cells. A CHC22 wild-type construct was made resistant to siCHC22.2 (construct vii, Fig. 2) by site-directed mutagenesis and stably introduced into SH-SY5Y cells. Treatment of these cells with siRNA against endogenous *CLTCL1* failed to induce significant neurite outgrowth indicating rescue of the knockdown induced phenotype (Fig. 6F and G).

In SH-SY5Y cells, differentiation with retinoic acid is accompanied by an upregulation of TRKB that is essential for induction of differentiation (Fig. 5C and 6E) (Esposito *et al.*, 2008). Therefore, we assessed whether CHC22 was involved in the vesicular trafficking of TRKB in differentiating SH-SY5Y cells and found no evidence of this. Furthermore, while treatment with retinoic acid caused a reduction in CHC22 accompanied by TRKB upregulation, knockdown of *CLTCL1* induced a similar morphological phenotype but with no change in TRKB levels. This indicates that CHC22 loss induces differentiation in a TRKB-independent manner (Fig. 6E).

### p.E330K renders CHC22 unable to negatively regulate neurite outgrowth

Next, we sought to confirm pathogenicity of the p.E330K mutation by testing whether it affected the function of CHC22 in neuronal differentiation. We manufactured a construct bearing the familial CHC22 mutation that was resistant to knockdown (construct viii; Fig. 2) and again made stable SH-SY5Y cell lines. In these cells, despite expression of CHC22.E330K, knockdown of endogenous CHC22 induced neurite outgrowth, indistinguishable from the effects observed in untransfected parental cells (Fig. 6F and G). These data further confirm the induction of neurite outgrowth to be a CHC22-specific effect, that CHC22 is an essential negative regulator of sensory neuron differentiation, and that the p.E330K mutation renders CHC22 unable to function in this process.

### CHC22 downregulation is an essential aspect of retinoic acid-mediated differentiation

**Figure 7:**
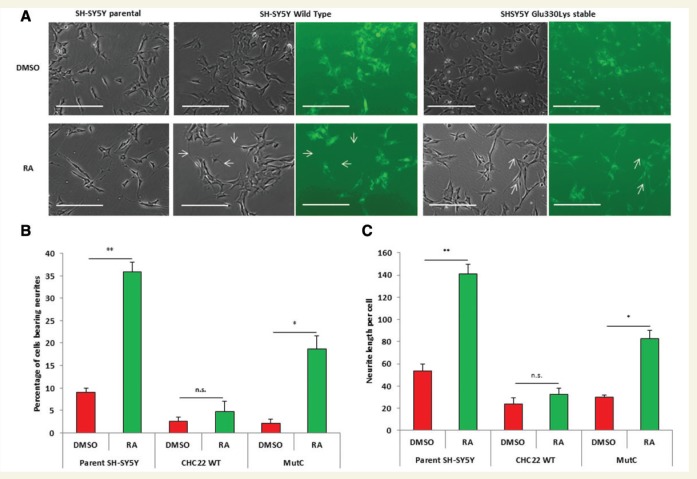
**Downregulation of CHC22 is essential for retinoic acid-mediated differentiation**. (**A**) Representative images taken on Day 5 with or without treatment with 10 µM retinoic acid (RA). Addition of retinoic acid to parental cells results in significant neurite outgrowth, whereas stable expression of CHC22.WT.GFP blocked this process. Note white arrows indicating non-transfected cells undergoing differentiation, but no evidence in cells expressing GFP. Stable expression of CHC22.E330K.GFP in SH-SY5Y cells are still able to be differentiated by addition of retinoic acid. Note white arrows indicating differentiated cells which do express GFP. (**B**) Quantification of percentage of cells bearing a neurite (greater than two cell bodies in length). (**C**) Quantification of the average neurite length per cell under each experimental condition. Data represents at least three independent experiments. Statistical tests were Student’s *t*-test: *P < 0.05 and **P < 0.01. Error bars represent standard error.

Having demonstrated that CHC22 overexpression could rescue the differentiation phenotype associated with siRNA-mediated knockdown of CHC22, we proceeded to investigate if downregulation of CHC22 was essential for differentiation by retinoic acid treatment. We took SH-SY5Y cells stably overexpressing CHC22 WT.GFP or CHC22 p.Glu330Lys.GFP and parental cells as a control, and treated them with 10 µM retinoic acid for 5 days. After 5 days we imaged the cells (Fig. 6A) and quantified neurite outgrowth. Although parental cells showed a significant increase in both the percentage of cells bearing a neurite greater than two cell bodies in length, and in the total neurite length per cell, cells expressing wild-type CHC22 were resistant to neurite outgrowth induction by retinoic acid (Fig. 7B and C). As expected, cells expressing the CHC22 mutant protein remained able to undergo differentiation, in similar proportion to that of parental cells (Fig. 7B and C).

## Discussion

We report the first cases of a novel syndrome of absent pain and touch sensing with cognitive delay. Genetic mapping and exome sequencing identified the potential causative mutation to reside in the *CLTCL1* gene encoding CHC22, the second clathrin heavy chain protein. The common clathrin heavy chain, CHC17 is ubiquitously expressed, whereas CHC22 displays a more restricted expression pattern. The functional unit of clathrin, known as a triskelion, is composed of three trimerized heavy chain subunits and three light chain subunits. Triskelia self-assemble on membranes forming a regular, polyhedral lattice associated with the formation of endosomes (Brodsky, 2012). The CHC17 protein functions in numerous endosomal trafficking pathways including receptor-mediated endocytosis and endosomal sorting at the trans-golgi network. However, the role of CHC22 is less well established but has been demonstrated to have a non-redundant role in the intracellular trafficking of the GLUT4 glucose transporter (encoded by *SLC2A4*) in postnatal muscle and fat tissue (Vassilopoulos *et al.*, 2009). Despite significant structural similarity, CHC22 and CHC17 seem to functional similarly only when CHC22 is expressed at higher than physiological levels (Esk *et al.*, 2010). Here we identify an additional, non-redundant role for CHC22 in the development of nociceptive and mechanoreceptive neurons.

We found our family’s mutation to be pathogenic in an *in vitro* assay of receptor-mediated endocytosis at the cell membrane, albeit at non-physiological levels where CHC22.WT or CHC22.E330K were overexpressed by transfection and with CHC17 simultaneously knocked down. In the presence of CHC22 at physiological levels and absence of CHC17, transferrin internalization is blocked thus confirming that CHC22 alone cannot induce clathrin-mediated endocytosis in HEK293 cells. We therefore propose that CHC22 is likely to possess a distinct function to that of CHC17 when expressed at normal cellular levels.

Previous studies have shown that CHC17 is much more abundant than CHC22, and that after birth CHC22 is expressed at higher levels in muscle cells where it functions in a cell-specific role in the formation of the GLUT4 storage compartment (Vassilopoulos *et al.*, 2009). We hypothesized that were CHC22 to also play a cell-specific role in neurogenesis, it would also likely be expressed at higher levels in the developing brain and that its expression pattern might mimic that of other known neurodevelopmental genes showing increase until mid-foetal development, and then gradual decline with age until early childhood (Kang *et al.*, 2011; Tebbenkamp *et al.*, 2014). Re-analysing data from the ‘Brainspan Atlas of the Developing Human Brain’, we found that in all regions of the cortex tested and in the hippocampus and striatum, *CLTCL1* was significantly increased during early prenatal development, dropping by as much as 7-fold by early childhood. This was in contrast to the expression of *CLTC* whose level is maintained throughout the course of neurodevelopment. This gene expression trajectory strongly suggests that CHC22 functions in neurodevelopment, potentially affecting neural progenitors or immature neurons which are known to develop at the time of peak *CLTCL1* expression (Tebbenkamp *et al.*, 2014).

To our surprise we found that during the development of human neural crest-derived cell systems for TRKA/pain and TRKB/touch neuron production, CHC22 is downregulated, and that downregulation of CHC22 by siRNA actively induces the differentiation of neural precursors. The inability to induce this effect in cells stably expressing CHC22 demonstrates that CHC22 has to be lost for development to progress. In contrast, persistent expression of mutant CHC22 was unable to rescue knockdown of endogenous CHC22 in this process, thus further confirming pathogenicity of the p.E330K mutation in *CLTCL1* and suggesting the pathogenic mechanism for the mutation in our family. We conclude that CHC22 is an essential negative regulator for the subset of neural crest progenitors that produce pain and touch sensing neurons.

*CLTCL1* derived from a duplication of *CLTC* coincident with the emergence of vertebrates, an animal subphylum defined by the possession of a spinal column and neural crest (Wakeham *et al.*, 2005). Notwithstanding selective pressures on CHC22 due to its function in postnatal, vertebrate muscle cells, it is conceivable that during development, the original clathrin heavy chain might maintain a generalized endosomal function but the duplicated clathrin heavy chain adopt a role restricted to the newly emerged vertebrate nervous system. We have not investigated the specific cerebral cause of the cognitive disability in this family, but note that CHC22 has been previously linked to cerebral behavioural phenotypes (Chahrour *et al.*, 2012).

There are two corollaries from our studies. Firstly, rodents have lost *CLTCL1* and thus must have alternative pathway(s) to compensate for this. Thus, some pain research results generated in these animals may not be applicable to man. Secondly, new classes of analgesics have resulted from studying known Mendelian causes of painlessness e.g. the developmental genes *NTRK1* and *NGF* (Holmes, 2012)—hopefully, similar advances will emerge from the further study of CHC22 and the developmental pathway it controls.

## Web resources

Human Genome Browser is at http://genome.ucsc.edu/. Primer3web is at http://bioinfo.ut.ee/primer3/. The 1000 genomes project is at http://browser.1000genomes.org/index.html. dbSNP is at www.ncbi.nlm.nih.gov/projects/SNP/. Integrated Genome Viewer is available from https://www.broadinstitute.org/igv/home. 1000 Genomes is at http://browser.1000genomes.org/index.html
